# Role of T cell—glial cell interactions in creating and amplifying central nervous system inflammation and multiple sclerosis disease symptoms

**DOI:** 10.3389/fncel.2015.00295

**Published:** 2015-08-05

**Authors:** Eric S. Huseby, Daisuke Kamimura, Yasunobu Arima, Caitlin S. Parello, Katsuhiro Sasaki, Masaaki Murakami

**Affiliations:** ^1^Department of Pathology, University of Massachusetts Medical SchoolWorcester, MA, USA; ^2^Division of Molecular Neuroimmunology, Institute for Genetic Medicine and Graduate School of Medicine, Hokkaido UniversitySapporo, Japan

**Keywords:** T cell, autoimmunity, glial fibrillary acidic protein, multiple sclerosis, astrocytes, experimental autoimmune encephalomyelitis, cerebellum

## Abstract

Multiple Sclerosis (MS) is an inflammatory disease of the Central Nervous System (CNS) that causes the demyelination of nerve cells and destroys oligodendrocytes, neurons and axons. Historically, MS has been thought of as a T cell-mediated autoimmune disease of CNS white matter. However, recent studies have identified gray matter lesions in MS patients, suggesting that CNS antigens other than myelin proteins may be involved during the MS disease process. We have recently found that T cells targeting astrocyte-specific antigens can drive unique aspects of inflammatory CNS autoimmunity, including the targeting of gray matter and white matter of the brain and inducing heterogeneous clinical disease courses. In addition to being a target of T cells, astrocytes play a critical role in propagating the inflammatory response within the CNS induced NF-κB signaling. Here, we will discuss the pathophysiology of CNS inflammation mediated by T cell—glial cell interactions and its contributions to CNS autoimmunity.

## Myelin-Specific T Cell Responses in MS and EAE

Multiple Sclerosis (MS), an inflammatory T cell-mediated autoimmune disease, is the most common neurological disease of young adults. MS causes the demyelination of nerve cells and destroys oligodendrocytes, neurons and axons (Frohman et al., 2006; Lassmann et al., 2007), with highly variable clinical manifestations. Such clinical manifestations of MS often include hyperreflexia, ataxia, spasticity and visual defects (Noseworthy et al., 2000; Keegan and Noseworthy, 2002; Hafler et al., 2005; Frohman et al., 2006; McFarland and Martin, 2007), and in some cases there are sensory defects and partial or complete paralysis. In the majority of patients, disease manifests as relapsing-remitting cycles of impairment, usually converting over time to a chronic progressive stage; 10–15% of patients present with disease that is progressive from onset (Sospedra and Martin, 2005; Frohman et al., 2006; McFarland and Martin, 2007; Steinman, 2009).

MS is thought to be primarily a CD4 T cell-mediated disease. Susceptibility to MS is genetically linked to major histocompatibility complex (MHC) genes and genes associated with T cell activation and homeostasis; however, the strongest genetic linkage occurs with certain alleles of MHC class II, which suggests a direct relationship between autoreactive CD4^+^ T cells and MS disease development in humans (Hillert and Olerup, 1993; Fogdell-Hahn et al., 2000; Sospedra and Martin, 2005). CD4^+^ T cells, in particular those that secrete IL-17, are considered to play an important role in the induction of central nervous system (CNS) autoimmunity (Korn et al., 2009). The identification of genes involved in CD4 T-cell differentiation and activation through genome wide association studies (GWAS) have further supported a role for CD4 T cells in the pathogenesis of MS (Patsopoulos et al., 2011).

The ability of myelin-reactive CD4 T cells to cause experimental autoimmune encephalomyelitis (EAE) further supports the hypothesis that myelin-reactive CD4 T cells have a central role in MS disease pathogenesis (Kuchroo et al., 2002; Sospedra and Martin, 2005; Ercolini and Miller, 2006; Hafler et al., 2007; Goverman, 2009; Steinman, 2009). MS-like clinical symptoms can be induced in animals by immunization with CNS proteins, as well as peptides derived from these CNS proteins, including myelin basic protein (MBP), proteolipid protein (PLP) and myelin oligodendrocyte glycoprotein (MOG; Ben-Nun et al., 2014). In addition, the adoptive transfer of activated CNS protein-specific CD4 T cells into naïve mice can induce paralytic diseases, allowing for *in vivo* study of the migratory behavior of pathogenic T cells (Jäger et al., 2009; Arima et al., 2012; Odoardi et al., 2012). However, it is unlikely that CD4 T cells are the sole mediators of disease pathogenicity, as treatments specifically targeting these cells limit neither the rate of disease relapses nor the formation of new lesions. In contrast, therapies that deplete or inhibit CNS infiltration of all lymphocyte subsets have been more successful (Lindsey et al., 1994; van Oosten et al., 1996; Rice et al., 2005).

Accumulating evidence strongly suggests that CD8 T cells also contribute to MS disease. Studies have shown that CD8 T cells are found in MS plaques—these cells are often oligoclonal, accumulate over time and can outnumber CD4 T cells regardless of the stage of activity or disease (Booss et al., 1983; Traugott et al., 1983; Hauser et al., 1986; Babbe et al., 2000; Lucchinetti et al., 2000; Frohman et al., 2006; Lassmann et al., 2007; Huseby et al., 2012). Though the antigen specificity of CNS infiltrating CD8 T cells remains unclear, a role for CD8 T cells in MS is further supported by the finding that particular MHC class I alleles can contribute to disease susceptibility (Cree et al., 2010; Healy et al., 2010).

Both a pathogenic or protective role for CNS-infiltrating CD8 T cells has been proposed. Myelin-specific CD8 T cells that are capable of killing neuronal cells *in vitro* have been isolated from MS patients (Tsuchida et al., 1994; Dressel et al., 1997; Medana et al., 2001; Crawford et al., 2004; Zang et al., 2004), which supports the hypothesis that CD8 T cells play a pathogenic role in the MS disease process. Further in support of this hypothesis, CD8 T cells specific for myelin proteins, including MBP, MOG, and PLP, have been shown to be pathogenic in several animal models of CNS disease (Huseby et al., 2001a; Sun et al., 2001; Ford and Evavold, 2005; Friese et al., 2008; Anderson et al., 2012). The clinical symptoms induced by such CNS-reactive CD8 T cells can be diverse. For example, mice carrying activated MBP-specific CD8 T cells succumb to a non-paralytic, acute demyelinating CNS autoimmunity that is clinically and histologically different than those of classic CD4-EAE. These atypical-EAE disease pathologies have similarities to MS patients with upper motor neuron disease (Huseby et al., 2001a). In contrast, experiments with MOG- and PLP-specific CD8 T cells resulted in CNS disease symptoms similar to classical EAE (Sun et al., 2001; Ford and Evavold, 2005; Friese et al., 2008; Anderson et al., 2012). These data suggest that myelin-specific CD8 T cells may contribute to some of the disease heterogeneity observed in MS patients.

Conversely, other studies have suggested that CD8 T cells may be suppressive during the MS disease process. CD8 T cell clones that can lyse myelin-specific CD4 T cells have been detected in MS patients (Chou et al., 1992; Zhang et al., 1993; Correale et al., 2000), and longitudinal magnetic resonance imaging (MRI) analysis has shown a negative correlation between the percentage of Tc2 cytokine-producing CD8 T cells in the periphery of MS patients and the development of lesions (Killestein et al., 2003). Moreover, protective MHC class I alleles have been identified through GWA studies, suggesting a relationship between autoreactive regulatory CD8^+^ T cells and MS disease development (International Multiple Sclerosis Genetics Consortium et al., 2011). In animal models, early studies found that polyclonal CD8 T cells can limit disease severity and relapses of CD4 T cell-mediated EAE (Jiang et al., 1992; Koh et al., 1992). The ability of CD8 T cells to regulate CNS autoimmune disease may occur by CD8 T cells targeting activated CD4 T cells through the recognition of peptide displayed on MHC class I and Ib molecules, as well as by secreting IL-10 and other anti-inflammatory soluble mediators (Jiang and Chess, 2006; Goverman, 2009; Kim and Cantor, 2011; Ortega et al., 2013). Thus, different subsets of CD8 T cells, like their CD4 counterparts, likely play pathogenic and immuno-regulatory roles in MS (Huseby et al., 2012).

## Gray Matter Lesions in MS and EAE

MS has traditionally been thought of as a disease that targets myelin proteins within the white matter of the CNS. Recent findings indicate, however, that this may not always be the case. Using advanced MRI techniques, multiple investigators have identified gray matter lesions in MS patients that appear at the earliest stages of disease and accumulate over time (Lucchinetti et al., 2000, 2011; Peterson et al., 2001; Bo et al., 2003; Frohman et al., 2006; Calabrese et al., 2007; Lassmann et al., 2007; Fisher et al., 2008; Ontaneda et al., 2012). The presence of T cells within gray matter lesions of MS patients suggests that T cells reactive to antigens other than myelin proteins may contribute to MS disease progression. One potential cellular target of gray matter disease is astrocytes, which reside within the white and gray matter of the CNS. Astrocytes normally express low levels of MHC, however levels increase during inflammation (Wong et al., 1984; Ransohoff and Estes, 1991; De Keyser et al., 2010).

Autoreactive T cells must avoid negative selection within the thymus and be exported to the peripheral T cell repertoire in order to contribute to the CNS autoimmune disease process. Though myelin proteins, the prototypical targets of encephalogenic CD4 T cells, are primarily expressed behind the blood-brain barrier, some myelin peptide epitopes are expressed and presented in the thymus. Developing T cells that are reactive to these ligands can be subject to thymic deletion or be skewed towards low avidity or suppressive responses. These findings have lead to a differential avidity model for the development of encephalogenic T cells: strong avidity T cells targeting myelin epitopes that are presented in the thymus undergo negative selection whereas weak avidity T cells that target these same epitopes or strong avidity T cells that target myelin epitopes that are only expressed within the CNS are exported into the mature T cell repertoire and can induce autoimmunity (Liu et al., 1995; Harrington et al., 1998; Targoni and Lehmann, 1998; Huseby et al., 1999, 2001b; Klein et al., 2000; Kuchroo et al., 2002). The expectation is that T cells which target astrocytes or other CNS cell types will follow similar rules for development as those identified for T cells that target myelin.

Two proteins predominately expressed in astrocytes, Glial fibrillary acidic protein (GFAP) and S100β, have been studied as targets for autoreactive T cells. GFAP, an intermediate filament protein, is an archetypal astrocyte-specific antigen that is expressed throughout the gray matter and white matter of the brain and spinal cord (Middeldorp and Hol, 2011). GFAP is also expressed in some peripheral tissues including the thymus, intestine and pancreas, though expression levels are lower in these tissue types (Zelenika et al., 1995). In MS lesions, the expression level of GFAP increases and peptides derived from GFAP are presented by MHC class I and class II molecules (Nait-Oumesmar et al., 2007; Fissolo et al., 2009; Linker et al., 2009). S100β, a calcium binding protein, is also expressed within astroglia present within the gray and white matter of the CNS (Zimmer et al., 1995). Although both proteins are also expressed outside of the CNS, including at a low level within the thymus, T cell responses to these proteins indicate that immune tolerance towards these antigens is incomplete.

The adoptive transfer of CD4^+^ T cells reactive to GFAP or to S100β into rodents induces a strong inflammatory response within the spinal cord and throughout the entire CNS, including the cerebral cortex and the retina of the eye, with particularly severe inflammation observed in the gray matter (Kojima et al., 1994, 1997). These experiments demonstrate that T cell responses to non-myelin antigens are capable of being pathogenic in models of CNS autoimmunity. Compellingly, CD4^+^ S100β-specific T cells have been isolated from MS patients, as well as from healthy controls, indicating astrocyte-specific T cells are present in the mature T cell repertoire and may contribute to the disease process (Schmidt et al., 1997).

## GFAP-Specific CD8 T Cells can Induce Relapsing/Remitting CNS Autoimmunity

The observation that CD8 T cells are present within gray matter lesions of MS patients (Peterson et al., 2001; Bo et al., 2003; Calabrese et al., 2007; Lassmann et al., 2007; Fisher et al., 2008; Lucchinetti et al., 2011; Ontaneda et al., 2012) inspired us to study astrocyte-specific CD8 T cells. We chose the astrocyte protein GFAP as the target antigen because GFAP expression and GFAP-peptide presentation by MHC class I and II molecules are increased within MS lesions (Nait-Oumesmar et al., 2007; Fissolo et al., 2009; Linker et al., 2009). Furthermore, although GFAP-specific T cells isolated from MS patients have not been studied, GFAP-specific CD8 T cells have been isolated from patients with type 1 diabetes, indicating that human T cells with this reactivity pattern populate the peripheral T cell repertoire (Standifer et al., 2006). CD8 T cells that target astrocytes and neurons have also been suggested in Rasmussen encephalitis (Schwab et al., 2009).

**Figure 1:**
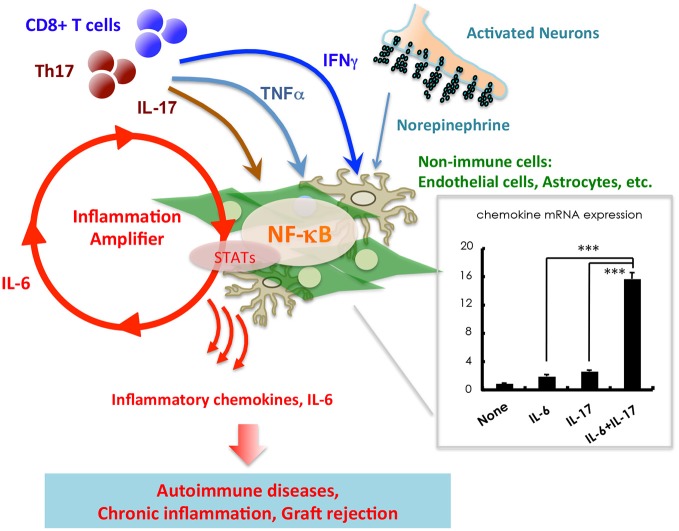
**Stimulation of non-immune cells including endothelial cells and astrocytes with IL-17, TNF*α*, IFN*γ*, and IL-6 from T cells induces a synergistic effect on the production of inflammatory chemokines such as CCL20 and IL-6**. An imaginary figure is shown. The synergistic effect requires the simultaneous activation of two transcription factors, NF-κB and STATs, in non-immune cells. Various soluble factors, including neurotransmitters from activated neurons, augment the inflammation amplifier by activating or sustaining the activation of NF-κB and STATs. Mean ± SD are shown. ***p < 0.001.

We have recently found that C57BL/6 mice carry CD8 T cells reactive to GFAP_264–272_ presented by H2-D^b^. We constructed TCR Tg mice expressing the GFAP-specific CD8 T cell clone, BG1 (BG1 mice), to follow the fate of naïve GFAP-specific T cells. To determine if BG1 mice maintain quiescence to GFAP over their lifetime, a cohort of WT, Rag1^−/−^ and *Gfap*^−/−^ BG1 mice were analyzed for clinical signs of CNS disease as they aged. We observed that BG1 mice do not maintain ignorance of GFAP: ~50% of WT BG1 mice and 100% of Rag^−/−^ BG1 mice succumb to spontaneous clinical signs of CNS autoimmunity by 6 months of age. The majority of diseased BG1 mice develop balancing defects, lethargy, uneven gait and ataxia—such symptoms are referred to as atypical disease (Sasaki et al., 2014)—whereas some diseased mice also succumb to mild ascending flaccid paralysis—such symptoms are referred to as classical EAE (Stromnes and Goverman, 2006). The atypical disease symptoms that develop in BG1 mice reflect the locations within the CNS that is targeted; BG1 mice develop lesions showing prominent glial responses within the cerebellum, mid-brain and spinal cord early in a spontaneous disease course that includes both white matter and gray matter (Figure 1).

The BG1 CD8 effector T cell populations that target the CNS during spontaneous CNS disease phenotypically resemble anti-viral tissue-resident memory (T_RM_) cells that populate peripheral tissues following viral challenges (Schenkel and Masopust, 2014). Functionally, only low frequencies of CD8 T cells within the CNS are capable of producing IFNγ, IL-17 or granzyme B (GZB), indicating that many of the BG1 CD8 T cells present within the brain are not classic effector CD8 T cells. These data suggest that BG1 CD8 T cells that spontaneously enter into the brain interact with astrocytes to induce their differentiation into auto-reactive T_RM_, without gaining inflammatory cytokine expression or cytotoxic effector functions. Nevertheless, these auto-reactive T_RM_ CD8 T cells can induce severe inflammation, glial responses and clinical disease symptoms. In contrast to CNS disease induced by auto-reactive T_RM_ CD8 T cells, disease induced by classic IFNγ-producing pro-inflammatory CD8 T cells demonstrates severe ataxia and lethargy within 7 days, a disease pattern highly similar to those induced by *in vitro* or Vac-activated MBP-specific CD8 T cells (Huseby et al., 2001a; Sasaki et al., 2014). These differences in CNS disease pathologies suggest that different auto-reactive CD8 T cell lineages induce distinct CNS disease phenotypes, thereby contributing to MS disease heterogeneity. This hypothesis is consistent with studies of encephalogenic CD4 T cells. Through the observation of CD4 T cells responding to different neuroantigens and different priming protocols, it has been demonstrated that the effector lineage and activation status of CD4 T cells within the CNS influence the location of lesions within the CNS, the severity of the acute disease as well as the overall clinical outcome (Kawakami et al., 2004; Jäger et al., 2009; Pierson et al., 2012).

In both WT and Rag1^−/−^ BG1 mice, spontaneous clinical symptoms begin as episodic bouts of functional impairment, with many mice displaying severe CNS dysfunction and then remitting to unobservable clinical symptoms. Rag1^−/−^ BG1 mice, however, develop more severe bouts of disease, and have more relapses than WT BG1 mice, with the majority progressing to a chronic disease stage. The observed differences in the frequency and severity of spontaneous disease between WT BG1 and Rag1^−/−^ BG1 mice suggests that GFAP-specific CD8 T cells are subject to extrinsic sources of immune regulation. To genetically map the lymphocytes that regulate GFAP-specific CD8 T cells, IA^b^β^−/−^ (MHC II-deficient) and μMT^−/−^ (B cell-deficient) BG1 mice were generated. Spontaneous CNS disease in IA^b^β^−/−^ BG1 mice was similar in frequency and severity to WT BG1 mice. In contrast, μMT^−/−^ BG1 mice were found to be highly susceptible to spontaneous CNS disease, with ~80% of μMT^−/−^ BG1 mice developing chronic clinical disease, a fundamentally distinct disease course as compared to the relapsing-remitting disease most often observed in WT BG1 and Rag^−/−^ BG1 mice (Sasaki et al., 2014). Thus, GFAP-specific CD8 T cell-mediated spontaneous relapsing-remitting and chronic disease is associated with the infiltration of tissue resident memory-like CD8 T cells into the CNS parenchyma and is regulated by polyclonal B cells. How B cells regulate CD8 T cell CNS autoimmunity, inflammation and disease remission is currently unknown.

## Does the Inflammation Amplifier Regulate Relapsing/Remitting CNS Disease?

In addition to immune cells, we have demonstrated that non-immune cells, including vascular endothelial cells and glial cells, play critical roles in the induction of chronic inflammatory diseases such as EAE. Glial cells of the CNS can secrete large quantities of chemokines, growth factors and IL-6 in response to inflammatory stimuli, all of which can activate the NF-κB and STAT signaling pathways (Ogura et al., 2008; Atsumi et al., 2014). This induction of inflammation, mediated by IL-17, TNFα, IFNγ, IL-6 or various neurotransmitters, is synergistically enhanced when both the NF-κB and STAT signaling pathways are induced in glial cells. We termed this synergistic effect the inflammation amplifier (Atsumi et al., 2014). Importantly, clinical symptoms of EAE, and several additional chronic inflammatory diseases, are significantly improved in mice unable to activate the inflammation amplifier (Ogura et al., 2008; Arima et al., 2012; Lee et al., 2012; Murakami et al., 2013; Harada et al., 2015). These findings indicate that the inflammation amplifier has a central role in chronic inflammatory diseases.

The inflammation amplifier is regulated by the production of several neurotransmitters, including norepinephrine and ATP. These findings led us to hypothesize that the inflammation amplifier may link the onset and severity of CNS diseases to mental and physical stress. Indeed, regional neural activity created by gravity of the Earth on the soleus muscles enhances chemokine expressions within the CNS, resulting in inflammation occurring around the dorsal vessels of the fifth lumbar cord, during early stages of EAE. CNS inflammation and the upregulation of chemokine expression can similarly be induced artificially using electric stimulation of peripheral muscles, formally demonstrating that neuronal activity can regulate the inflammation amplifier *in vivo* (Arima et al., 2012). These phenomena have been termed the “gateway reflex” as these neural stimulations can create “gateways” for immune cells to enter into the CNS (Kamimura et al., 2013; Sabharwal et al., 2014).

## Future Studies

The inflammation amplifier can be turned on or off in response to acute inflammation, as well as to mental and physical stress. Thus, the temporal regulation of NF-κB and STAT signaling pathways in glial cells may regulate episodic cycles of relapsing/remitting clinical disease in MS patients. Mechanistically, one way this may occur is by recruiting or limiting immune cell migration through the “gateway” present within the spinal cord and potentially through other sites within the brain. Clarifying these mechanisms, and identifying how different immune cell lineages and subsets respond to and regulate the inflammation amplifier, will provide insights into the pathogenesis of relapsing/remitting CNS diseases, and identify drug-targetable molecular pathways that can be exploited to minimize MS disease relapses.

## Conflict of Interest Statement

The authors declare that the research was conducted in the absence of any commercial or financial relationships that could be construed as a potential conflict of interest.
